# Association between frailty syndrome and survival in patients with pancreatic adenocarcinoma

**DOI:** 10.1002/cam4.2157

**Published:** 2019-04-29

**Authors:** An Ngo‐Huang, Holly M. Holmes, Jude K. A. des Bordes, Nathan H. Parker, David Fogelman, Maria Q. B. Petzel, Juhee Song, Eduardo Bruera, Matthew H. G. Katz

**Affiliations:** ^1^ Department of Palliative, Rehabilitation, and Integrative Medicine The University of Texas MD Anderson Cancer Center Houston Texas; ^2^ Division of Geriatric and Palliative Medicine, McGovern Medical School The University of Texas Health Science Center Houston Texas; ^3^ Department of General Internal Medicine The University of Texas MD Anderson Cancer Center Houston Texas; ^4^ Department of Behavioral Science The University of Texas MD Anderson Cancer Center Houston Texas; ^5^ Department of Surgical Oncology The University of Texas MD Anderson Cancer Center Houston Texas; ^6^ Department of Gastrointestinal Medical Oncology The University of Texas MD Anderson Cancer Center Houston Texas; ^7^ Department of Biostatistics The University of Texas MD Anderson Cancer Center Houston Texas

**Keywords:** cancer survival, frailty, geriatric assessment, geriatric syndromes, pancreatic adenocarcinoma

## Abstract

**Background:**

Frailty is a syndrome characterized by weakness, slow gait, weight loss, exhaustion, and low activity. We sought to determine whether frailty was associated with age or stage in newly diagnosed patients with pancreatic ductal adenocarcinoma (PDAC), and determine its association with survival.

**Methods:**

Consecutive patients with newly diagnosed PDAC of all stages underwent baseline assessment. Frailty (per Fried criteria) was defined as having three or more abnormalities in: grip strength, gait speed, weight loss, self‐reported exhaustion, or self‐reported physical activity. Baseline clinicodemographic characteristics, anatomic stage, performance status, and laboratory markers of prognosis were included. The association between baseline characteristics, frailty, and survival was determined. The associations of individual frailty measures with age, stage, comorbidities, and performance status were examined. Body composition was measured from computed tomographic images using SliceOMatic software.

**Results:**

Of 150 patients enrolled, 8 were excluded because they did not have PDAC on final diagnosis. The median age was 65 years (range, 32‐89). Seventy‐nine patients (55.6%) were sarcopenic, and 36 (25.4%) were frail. Frailty was associated with increasing comorbidities (*P* = 0.03) and worse performance status (*P* < 0.01). During follow‐up, 79 patients (56%) died. Frailty was significantly associated with death during the follow‐up period (*P* < 0.001) for the entire cohort, including patients with curative (*P* = 0.038) and palliative (*P* = 0.003) treatment plans.

**Conclusions:**

Frailty was seen frequently in patients with newly diagnosed PDAC and was not associated with increasing age or more advanced stage. Frailty was a predictor of survival, including patients treated with curative intent.

## 1 INTRODUCTION

Pancreatic cancer is typically a disease of older persons, with a median age at presentation of 71 years.^1^ Treatment that includes resection presents the only chance for survival. Older patients benefit from therapy that includes resection, but studies have historically shown higher risks of perioperative morbidity and mortality in this population. Although recent series have concluded that most older patients are able to receive curative therapy that includes resection, older patients who are actually selected for surgery may be among the fittest and are less likely to have common age‐related vulnerabilities.^2^, ^3^

After undergoing surgery for cancer, older persons are more susceptible to adverse outcomes including postoperative complications, discharge to a location other than home, and postoperative mortality. Assessment of age‐related vulnerabilities may aid in prognostication and in planning for the most appropriate therapy for older patients with cancer.^4^ Geriatric assessment is a multidimensional assessment of older patients that uses validated tools to assess medical, functional, physical, and psychosocial status^5^ and is recommended to determine optimal care for all older patients undergoing surgery.^6^ Individual tools used in geriatric assessment have been associated with perioperative complications, discharge location other than home, decline in functional status, longer length of hospital stay, and readmissions.^4^

Developing a tool for predicting adverse outcomes in pancreatic cancer is challenging, particularly since patients with newly diagnosed pancreatic cancer frequently present with cachexia and functional decline at baseline, a clinical presentation similar to the frailty syndrome commonly associated with advanced age. Frailty is characterized by poor nutrition, sarcopenia, and physical decline and has been associated with a higher risk of functional decline and mortality in older persons.^7^ Frailty has been used as a prognostic marker in older surgical candidates and is associated with increased risk of complications and mortality.^8^ A study of geriatric assessment in patients with pancreatic cancer who were surgical candidates found that self‐reported exhaustion (a component of the frailty syndrome) was independently associated with increased risk of major postoperative complications, postsurgical intensive care unit stay, and increased length of stay. Better scores on objective physical performance measures were associated with a lower likelihood of discharge to rehabilitation.^9^

We sought to determine the prognostic relevance of frailty (using Fried frailty criteria) in patients with newly diagnosed pancreatic ductal adenocarcinoma (PDAC), and to determine whether frailty was associated with increased age or more advanced stage of disease at presentation. We hypothesized that the presence of frailty at initial diagnosis would be associated with more advanced cancer stage, with the inability to complete curative therapy (ie, multimodality treatment including surgical resection), and with death within 1 year. Secondarily, we sought to explore whether frailty criteria were correlated with objective measures of muscle and fat mass from computed tomography (CT) scans, given that the availability of CT scans for patients with pancreatic cancer might facilitate routine integration of frailty status into clinical care. To test this hypothesis, we enrolled patients with newly diagnosed PDAC of all stages into a prospective cohort study to determine the impact of frailty on outcomes.

## 2 PATIENTS AND METHODS

### 2.1 Patient recruitment

This study was approved by The University of Texas MD Anderson Cancer Center Institutional Review Board (IRB #2012‐0318), and our research met the requirements for protection of human subjects. All patients were enrolled after obtaining written informed consent. Patients aged 18 years and older with newly diagnosed, previously untreated PDAC who presented to MD Anderson Cancer Center for treatment options were enrolled. Patients who did not speak English were excluded. Patients were enrolled in the Gastrointestinal Oncology Center. All data were collected during routine appointments with medical or surgical oncologists. We enrolled 150 consecutive consenting patients. One study investigator screened for potentially eligible patients, and a different study investigator conducted baseline assessments of patients, in order to provide blinding of the stage of pancreatic cancer at the time of frailty assessment.

### 2.2 Measures

Our primary objectives were (a) to determine the association of frailty with cancer stage or age, and (b) to determine the association of frailty at baseline with survival in patients with resectable cancer at presentation. A secondary objective was to determine whether frailty was significantly associated with radiographic measures of muscle and fat mass. We used the definition for a phenotype of frailty based on Fried criteria.^7^ Frailty was determined by the assessment of gait speed, grip strength, weight loss, exhaustion, and physical activity. Frailty was considered to be present if three or more assessments were abnormal, based on accepted cutoff values.

Gait speed was determined by a 3‐m timed walk, with abnormal gait speed being 3.62 seconds or longer to walk 3 m.^10^ Grip strength was measured by using a Jamar hydraulic hand dynamometer. Normal grip strength was determined by cutoffs used for men and women according to body mass index (BMI).^11^ Weight loss (by self‐report) was considered abnormal if a patient reported losing 3 kg or greater in the prior 3 months.^12^ Self‐reported exhaustion was determined by using two items from the Centers for Epidemiological Studies Depression (CES‐D) scale, and exhaustion was considered present if a patient reported feeling that “everything I did was an effort” or the patient “could not get going” for a moderate amount of time or for most of the time in the past week. Finally, physical activity was determined by using the International Physical Activity Questionnaire (IPAQ), which quantifies the amount of time in the prior 7 days that a person spent doing vigorous, moderate, or sedentary activity. The IPAQ was used to calculate the metabolic equivalent of task‐minutes per week for each patient; a value of 600 or less was considered to be low physical activity based on established physical activity guidelines.^13^

Selected demographic and clinical characteristics that were hypothesized to be associated with frailty were included as covariates. These included age, gender, race/ethnicity, BMI, and comorbidity. Comorbidity was determined by using the Adult Comorbidity Evaluation (ACE‐27), a 27‐item system‐based tool that categorizes comorbidity as none (0), mild (1), moderate (2), or severe (3).^14^ Anatomic stage of cancer at presentation was categorized as potentially resectable, borderline resectable, locally advanced, or metastatic, as determined by the primary oncology team based on radiographic data. The primary oncology team determined performance status at presentation, which was documented according to the Eastern Cooperative Oncology Group (ECOG) performance status scale. Performance status based on the Karnofsky Scale was converted to ECOG scores.^15^ Finally, baseline CA 19‐9 level, albumin, and total bilirubin were included as potential factors associated with cancer stage or with frailty. The initial treatment plan was determined to be curative in patients for whom surgical resection was planned, with or without chemotherapy and/or chemoradiation before or after surgery. Patients who were not surgical candidates were categorized as having a palliative plan of care on presentation.

Baseline anthropometric analyses included cross‐sectional areas of skeletal muscle, intramuscular fat, visceral fat, and subcutaneous fat from CT images of the abdomen and pelvis. Dicom images of the midpoint of the L3 vertebral body were analyzed with the use of SliceOMatic software (Tomo‐Vision, 2012). Cross‐sectional areas were standardized to the square of the height in meters. Radiographic evidence of sarcopenia was defined as skeletal muscle mass ≤38.9 cm^2^/m^2^ for women and ≤55.4 cm^2^/m^2^ for men.^16^

### 2.3 Statistical methods

For determining the association of frailty with cancer stage or age (objective 1), primary outcome comparisons were the associations between frailty and increasing age and between frailty and more advanced cancer stage. We used descriptive statistics for the prevalence of frailty and the prevalence of abnormalities on each frailty index assessment. We reported all baseline characteristics by patient status as not frail or frail. For categorical variables, we compared the proportion who were frail or not frail by using chi‐square tests or by Fisher exact tests. For ordinal variables with significant associations with frailty, the Cochran‐Armitage test for trend was used to confirm a significant trend in the association with frailty for increasing ordinal level. For continuous covariates, we used *t* tests for normally distributed variables and the Wilcoxon rank sum test for variables not distributed normally to compare differences by frailty status. Individual frailty measures were treated as categorical normal/abnormal results and analyzed in the same way as frailty.

To determine the association of frailty status at baseline with survival in patients with resectable cancer at presentation (objective 2), only patients with a curative plan of care at presentation were included in the analysis. Survival time is defined as the time interval from diagnosis to death, censored at last follow‐up.

Kaplan‐Meier graphs and Cox proportional hazards models for overall survival were evaluated for the presence of frailty, age, comorbidity, anatomic stage, and performance status, along with the receipt of any preoperative chemotherapy or curative resection (pancreaticoduodenectomy or pancreatectomy). The Firth correction was applied for Cox proportional hazards models to account for low cell numbers.

The association between frailty and measures of body composition was determined with use of *t* tests or with Wilcoxon rank sum tests when body composition variables were not normally distributed. All values for body composition were measured in centimeters squared and then corrected for body surface area.

Analyses were completed with the use of Stata 12 (StataCorp, College Station, TX); survival analyses also used SAS version 9.4 (SAS Institute, Cary, NC). All tests were two‐sided, and we considered *P* < 0.05 to be statistically significant.

## 3 RESULTS

**Figure 1:**
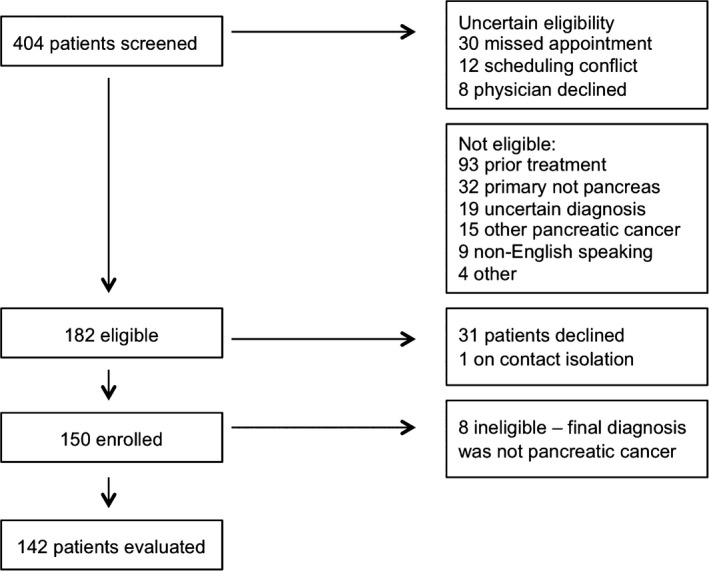
Study enrollment flow chart

We enrolled and completed assessments for 150 adult patients presenting to this quaternary cancer center for treatment options for a pancreatic mass. Eight patients did not have PDAC. We ultimately included 142 patients with newly diagnosed, previously untreated PDAC in our analysis. Figure 1 is a flow diagram for study recruitment. The mean and median age for all patients was 65 years, ranging from 32 to 89 years. For 47 patients, a curative plan that included surgery was recommended and the rest of the patients were offered palliative treatment.

Overall, 36 patients (25.4%, 95% confidence interval [CI], 18.1%‐32.6%) were frail. Table 1 shows the patient characteristics according to frailty category (nonfrail vs frail). Frailty was not associated with increasing age or with more advanced stage of cancer at presentation. Frailty was significantly associated with higher comorbidity scores, with a significant trend for frailty by increasing comorbidity (test for trend, *P* < 0.01). Frailty was also associated with worse ECOG performance status, with a higher prevalence of frailty by worse performance status (test for trend, *P* < 0.01). There was no significant association between frailty and sex, race, BMI, CA19‐9, albumin, bilirubin levels, or chemotherapy regimen.

**Table 1:** Patient characteristics (n = 142) by the presence of frailty at baseline and characteristics of curative patients only (n = 47)

Characteristic	Category	All patients n = 142, column%	Not frail n = 106 (74.6%), column%	Frail n = 36 (25.4%), column%	*P* value*	Curative patients n = 47, column%
Age (y)	18‐54	22 (15.5)	17 (16.0)	5 (13.9)	0.67	6 (12.8)
	55‐64	48 (33.8)	37 (34.9)	11 (30.6)		16 (34.0)
	65‐74	50 (35.2)	38 (35.9)	12 (33.3)		17 (36.2)
	75+	22 (15.5)	14 (13.2)	8 (22.2)		8 (17.0)
Sex	Men	93 (65.5)	74 (69.8)	19 (52.8)	0.06	34 (72.3)
	Women	49 (34.5)	32 (30.2)	17 (47.2)		13 (27.7)
Race/Ethnicity	White	119 (83.8)	91 (85.9)	28 (77.8)	0.06	40 (85.1)
	Black	8 (5.6)	6 (5.7)	2 (5.6)		2 (4.3)
	Hispanic	6 (4.2)	2 (1.9)	4 (11.1)		2 (4.3)
	Asian	5 (3.5)	5 (4.7)	0 (0)		2 (4.3)
	Other	4 (2.8)	2 (1.9)	2 (5.6)		1 (2.1)
BMI (kg/m^2^)	<25	49 (34.5)	39 (36.8)	10 (27.8)	0.32	17 (36.2)
	≥25	93 (65.5)	67 (63.2)	26 (72.2)		30 (63.8)
ACE‐27 Score	0	31 (21.8)	28 (26.4)	3 (8.3)	**0.03**	11 (23.4)
	1	54 (38.0)	42 (39.6)	12 (33.3)		20 (42.6)
	2	41 (28.9)	26 (24.5)	15 (41.7)		10 (21.3)
	3	16 (11.3)	10 (9.4)	6 (16.7)		6 (12.8)
Stage	PR	37 (26.1)	31 (29.3)	6 (16.7)	0.22	29 (61.7)
	BR	17 (12.0)	14 (13.2)	3 (8.3)		12 (25.5)
	LA	26 (18.3)	16 (15.1)	10 (27.8)		6 (12.8)
	Metastatic	62 (43.7)	45 (42.5)	17 (47.2)		0
ECOG PS	0	72 (50.7)	61 (57.6)	11 (30.6)	**<0.01**	36 (76.6)
	1	54 (38.0)	38 (35.9)	16 (44.4)		11 (23.4)
	2	12 (8.5)	7 (6.6)	5 (13.9)		0
	3	4 (2.8)	0 (0)	4 (11.1)		0
CA 19‐9, U/mL, median (IQR)		329 (1829)^a^	294 (1693)	556 (2887)	0.56	118.4 (152.2)^b^
Albumin, g/dL, mean (SD)		4.2 (0.44)^c^	4.2 (0.42)	4.1 (0.49)	0.11	4.2 (0.44)^d^
Bilirubin, mg/dL, median (IQR)		0.7 (0.95)^e^	0.7 (0.8)	0.8 (2.0)	0.39	1.3 (2.2)
Frail	No	106 (74.6)				40 (85.1)
	Yes	36 (25.4)				7 (14.9)
Grip strength	Normal	126 (88.7)	103 (97.2)	23 (63.9)	<0.001	42 (89.4)
	Abnormal	16 (11.3)	3 (2.8)	13 (36.1)		5 (10.6)
Gait speed	Normal	123 (86.6)	103 (97.2)	20 (55.6)	<0.001	45 (95.7)
	Abnormal	19 (13.4)	3 (2.8)	16 (44.4)		2 (4.3)
Weight loss	Normal	49 (34.5)	46 (43.4)	3 (8.3)	<0.001	16 (34.0)
	Abnormal	93 (65.5)	60 (56.6)	33 (91.7)		31 (66.0)
Physical activity	Normal	75 (52.8)	69 (65.1)	6 (16.7)	<0.001	29 (61.7)
	Abnormal	67 (47.2)	37 (34.9)	30 (83.3)		8 (38.3)
Exhaustion	Normal	97 (68.3)	93 (87.7)	4 (11.1)	<0.001	35 (74.5)
	Abnormal	45 (31.7)	13 (12.3)	32 (88.9)		12 (25.5)

Bold *P* values indicate statistical significance.

Abbreviations: ACE‐27, Adult Comorbidity Evaluation; BMI, body mass index; BR, borderline resectable; ECOG PS, Eastern Cooperative Oncology Group performance status; LA, locally advanced; PR, potentially resectable.

a Reported in 99 patients with CA 19‐9 levels available and with bilirubin levels <1.5 mg/dL

b Reported in 26 patients with CA 19‐9 levels available and with bilirubin levels <1.5 mg/dL

c n = 134, 8 missing values

d n = 46, 1 missing value

e n = 136, 6 missing values

* *P* value denotes comparison of not frail vs frail groups.

The most common abnormality on the frailty index was weight loss, which was abnormal among 65.5% of all patients. Most patients categorized as frail had weight loss (91.7%), exhaustion (88.9%), or low physical activity (83.3%). Few patients who were not frail had abnormal grip strength or gait speed; 81.3% of all patients who had abnormal grip strength were frail, 84.2% of all patients with abnormal gait speed were frail. Frail patients were more likely to have abnormalities in each of the five components of the Fried criteria for frailty (*P* < 0.001).

Also included in Table 1 are the characteristics of the subset of patients treated with curative intent and the corresponding frailty assessment results. Seven (14.9%) of the 47 curative patients were frail. Of the curative patients who were frail, weight loss (66%) was the most common abnormal component of frailty.

Although 30.1% of the patients recommended for palliative treatment were frail, compared with 14.9% of the curative patients who were frail, there was no significant association between frailty status and treatment plan at presentation (*P* = 0.11). Among patients treated with curative intent, frailty was not associated with surgical resection (*P* = 0.44).

Table 2 shows associations between components of Fried Criteria for frailty and age, ACE‐27 comorbidity score, cancer stage, and ECOG performance status. Older age was significantly associated with weak grip strength, slow gait speed, and weight loss. A worse comorbidity score was associated with weak grip and slow gait speed. Only disease stage was associated with self‐reported low physical activity. Worse ECOG performance status was associated with abnormalities in all components of the frailty index, except for self‐reported low physical activity.

**Table 2:** Number (row %) with abnormalities on individual frailty measures by age, stage, comorbidity, and ECOG for all patients (n = 142)

Characteristic	Category	Total number	Weak grip n = 16	*P* value	Slow gait n = 19	*P* value	Weight loss n = 93	*P* value	Low activity n = 67	*P* value	Exhaustion n = 45	*P* value
Age (y)	18‐54	22	0	**<0.01**	1 (4.6)	**<0.01**	12 (54.6)	<0.01*	12 (54.5)	0.83	7 (31.8)	0.78
	55‐64	48	2 (4.2)		3 (6.3)		39 (81.3)		23 (47.9)		14 (29.2)	
	65‐74	50	5 (10.0)		6 (12.0)		33 (66.0)		23 (46.0)		15 (30.0)	
	75+	22	9 (40.9)		9 (13.4)		9 (40.9)		9 (40.9)		9 (40.9)	
ACE‐27 Score	0	31	0	**0.02**	1 (3.2)	**<0.01**	20 (64.5)	0.97	13 (41.9)	0.54	7 (22.6)	0.6
	1	54	5 (9.3)		5 (9.3)		35 (64.8)		23 (42.6)		17 (31.5)	
	2	41	7 (17.1)		6 (14.6)		28 (68.3)		23 (56.1)		15 (36.6)	
	3	16	4 (25)		7 (43.8)		10 (62.5)		8 (50.0)		6 (37.5)	
Stage	PR	37	4 (10.8)	0.57	5 (13.5)	0.87	21 (56.8)	0.56	8 (21.6)	**<0.01**	9 (24.3)	0.11
	BR	17	1 (5.9)		1 (5.9)		11 (64.7)		12 (70.6)		3 (17.7)	
	LA	26	5 (19.2)		9 (34.6)		17 (65.4)		15 (57.7)		13 (50.0)	
	Metastatic	62	6 (9.7)		4 (6.5)		44 (71.0)		32 (51.6)		20 (32.3)	
ECOG PS	0	72	5 (6.9)	**<0.01**	4 (5.6)	**<0.01**	40 (55.6)	0.04*	29 (40.3)	0.1	15 (20.8)	**<0.01**
	1	54	6 (11.1)		7 (13.0)		41 (75.9)		28 (51.9)		20 (37.0)	
	2	12	2 (16.7)		4 (33.3)		10 (83.3)		6 (50.0)		6 (50.0)	
	3	4	3 (75.0)		4 (100)		2 (50.0)		4 (100)		4 (100)	

Fisher's tests used for cell counts <5. Bold *P* values indicate statistical significance.

Abbreviations: ACE‐27, Adult Comorbidity Evaluation; BR, borderline resectable; ECOG PS, Eastern Cooperative Oncology Group performance status; LA, locally advanced; PR, potentially resectable.

* Test for trend with increasing category was not significant.

Measures of body composition via CT determination of skeletal muscle and fat content at the L3 level were available for 134 patients (Table 3). Seventy‐nine patients (55.6%) met anthropometric criteria for sarcopenia,^16^ including 60 men (64.5%) and 19 women (38.8%). Overall, the frail population had significantly more subcutaneous fat compared with the nonfrail population (*P* = 0.02). Frail men had significantly more intramuscular fat than nonfrail men (*P* = 0.05). Surprisingly, frail women had significantly higher skeletal muscle mass than nonfrail women (*P* = 0.02). Of note, the frail women in this cohort had a higher BMI, on average, than did nonfrail women (30.2 vs 26.0, *P* = 0.06). There was no significant difference in BMI between men and women.

**Table 3:** Measures of body composition among patients with radiographic data according to the presence of frailty, by gender

Body composition measure^a^	Overall (mean ± SD)	Not frail (mean ± SD)	Frail (mean ± SD)	*P*value
	n = 134	n = 102	n = 32	
Muscle mass, n = 134	48.2 ± 9.0	48.2 ± 9.5	48.1 ± 7.4	1.0
Intramuscular fat, n = 134	4.7 ± 2.8	4.4 ± 2.4	5.7 ± 3.8	0.08
Visceral fat, n = 134	57.3 ± 45.8	55.9 ± 46.9	61.8 ± 42.5	0.53
Subcutaneous fat, n = 130	65.4 ± 31.5	61.9 ± 30.2	77.2 ± 33.4	**0.02**
Men	n = 88	n = 71	n = 17	
Muscle mass, n = 88	51.8 ± 8.1	51.9 ± 8.5	51.2 ± 6.2	0.77
Intramuscular fat, n = 88	4.6 ± 3.0	4.1 ± 2.2	6.5 ± 4.6	**0.05**
Visceral fat, n = 88	66.7 ± 50.2	63.5 ± 51.7	80.1 ± 41.6	0.22
Subcutaneous fat, n = 88	58.7 ± 25.5	56.3 ± 25.4	69.0 ± 24.0	0.06
Women	n = 46	n = 31	n = 15	
Muscle mass, n = 46	41.3 ± 6.5	39.7 ± 5.6	44.6 ± 7.2	**0.02**
Intramuscular fat, n = 46	5.0 ± 2.6	2.7 ± 0.5	2.4 ± 0.6	0.7
Visceral fat, n = 46	39.4 ± 28.8	26.6 ± 4.8	34.0 ± 8.8	0.79
Subcutaneous fat, n = 42	79.5 ± 38.0	36.5 ± 6.8^b^	41.4 ± 11.5^c^	0.35

Bold *P* values indicate statistical significance.

a All values are measures in cm^2^ divided by height in m^2^.

b n = 29, 2 missing values

c n = 13, 2 missing values

**Figure 2:**
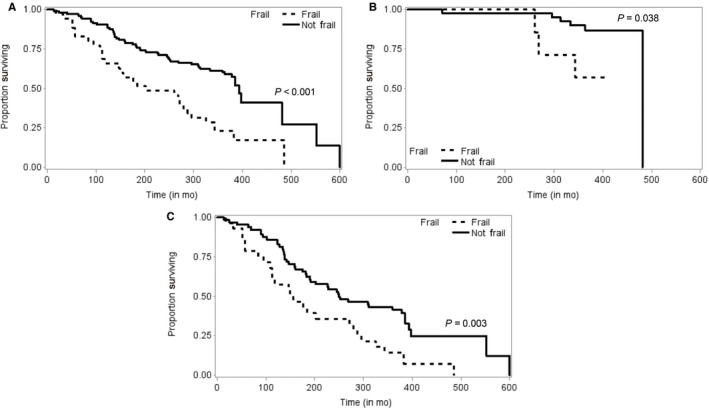
Kaplan‐Meier curves showing overall survival according to the initial treatment plan, by frailty status: (A) overall survival in all patients (n = 142) by frailty, (B) overall survival in curative patients (n = 47) by frailty, and (C) overall survival in palliative patients (n = 95) by frailty

Overall, 79 patients (56%) died during follow‐up; 4 patients were lost to follow‐up. Median overall survival from the date of diagnosis was 364 days (range, 14‐680 days). Frailty status was significantly associated with the increased risk of death (HR, 2.50, 95% CI, 1.57‐3.98, *P* < 0.001). Figure 2 shows the Kaplan‐Meier survival functions by frailty status for all patients and by subgroup analysis for those treated with curative intent and palliative intent. Frailty was significantly associated with death during the follow‐up period (*P* < 0.001) for the entire cohort, including patients with curative (*P* = 0.038) and palliative (*P* = 0.003) treatment plans. We conducted a subgroup analysis of the nonfrail group and found 85 patients (60%) were prefrail. There was no significant decrease in overall survival in those who were prefrail vs nonfrail (HR, 1.63, 95% CI 0.73‐3.64, *P* = 0.237).

## 4 DISCUSSION

Frailty and sarcopenia were quite frequent in this population of patients with PDAC who were not exclusively older patients. Frailty was not associated with advanced age or with worse cancer stage. However, frailty was associated with greater comorbidity and worse performance status at initial presentation. The presence of frailty did not preclude a curative treatment plan as 7 (14.9%) of the patients with a curative treatment regimen (including surgery) were frail. In the overall cohort of patients, frailty was associated with worse survival. This difference was significant among patients treated with palliative intent and was not significant among patients treated with curative intent. Our findings suggest that among patients with curative intent, other factors are more relevant to survival than frailty—thus the presence of frailty alone should not prohibit potentially curative therapy.

A systematic review conducted in 2016 on frailty in older surgical patients showed that frailty was associated with increased mortality, postoperative complications, prolonged length of hospital stay, and discharge to a nursing care facility. This review included operations performed for cancer and noncancer diagnoses and found that frailty was strongly associated with increased 30‐day, 90‐day, and 1‐year mortality.^17^ Dale et al^9^ used the geriatric assessment in patients undergoing pancreaticoduodectomy. The patient population included noncancer patients (28% with benign diagnoses) who had all undergone surgery. Their study found that older age was associated with decreased survival after pancreaticoduodectomy and was associated with the higher rates of morbidity and mortality. They did not evaluate the presence of frailty itself as a whole or its influence on survival. They found that the most common abnormal components in frail patients were unintentional weight loss (56%), weak grip strength (42%), and self‐reported exhaustion (37%); our study, however, found that weight loss, low physical activity, and self‐reported exhaustion were most common.

Certain variables in the Fried Criteria were significantly associated with prognosis. Exhaustion was the only frailty measure that was significantly associated with performance status. In addition, self‐reported low physical activity level was significantly associated with performance status, but not with age, comorbidities, or stage. Perhaps a more simplified frailty tool that does not include the components of exhaustion and self‐reported low physical activity level may be developed in the future since more objective measures such as grip strength, gait speed, and weight loss may have more prognostic value.

In this study, frailty was not significantly associated with age. Other studies involving the geriatric assessment and/or frailty measures have included older patients only or did not study the effects of age on frailty.^9^, ^18^, ^19^ This suggests that frailty may be present in younger patients with PDAC; thus, frailty assessments should be completed in younger patients since such assessments may help identify patients who would benefit from intervention.

We also examined the association of body composition with frailty. There was a high percentage of sarcopenic patients (55.6%) in this cohort, which is consistent with our previous studies on body composition before initiating treatment.^20^, ^21^ There was greater intramuscular fat and subcutaneous fat in frail men, but not in women. The frail women had significantly higher skeletal muscle mass than did the nonfrail women; however, the BMI of the frail women was also higher and thus would account for this unexpected result.

Frail patients had significantly decreased survival rates both among the entire cohort including the groups treated with curative and palliative intent. However, prefrail patients were not significantly different from nonfrail patients in overall survival. This contrasts the findings of Fried et al who found that prefrail or “intermediate frailty status” was associated with an intermediate risk of adverse outcomes, including death.^7^ Interventions targeted at reducing frailty have the potential to improve outcomes. For example, closer monitoring of weight and appetite symptoms should warrant closer follow‐up by a clinical dietitian. Interventions to increase physical activity may also prevent or reverse frailty. Our own group has demonstrated the feasibility of preoperative exercise during neoadjuvant chemotherapy and/or chemoradiation for patients with potentially resectable pancreatic cancer.^22^ Future studies will evaluate which types of prehabilitation interventions are effective in improving or reversing the frailty syndrome.

### 4.1 Limitations and implications

This study was conducted at a quaternary cancer center; patients who are able to travel from far distances are, in general, in more robust physical condition. Thus, although frailty was present in 26% of this cohort of patients, the presence of frailty in the general population of patients with newly diagnosed PDAC is likely higher.

Because certain components of the Fried Criteria have more prognostic value, it may be worthwhile to consider a frailty criterion specific to patients with PDAC. In the Fried Criteria, patient‐reported weight loss and physical activity were used. Because self‐reported physical activity may not be a sensitive measure of prognosis, objective measures of physical activity such as the use of wearable trackers may be use more useful. Future studies should evaluate the association of frailty on postoperative outcomes, specifically in patients with PDAC. A systematic review on frailty in older surgical patients found that the strongest association was between frailty and 30‐day mortality.^17^ Unfortunately, we did not evaluate postoperative outcomes in this cohort of patients due to the small number of frail patients in the curative treatment group. Although our findings support the use of frailty screening in patients with newly diagnosed PDAC, these assessments are not widely used to determine treatment plans. McCarthy et al^23^ used a frailty index to predict chemotherapy outcomes in older patients with cancer. They found that patients who were not assigned treatment were significantly more frail than were patients who completed chemotherapy or ones who discontinued chemotherapy prematurely. These findings, in conjunction with ours, suggest that frailty screens should not be an indication to preclude treatment. However, the presence of frailty denotes high level of vulnerability and these patients require more frequent follow‐up and aggressive monitoring by rehabilitation, supportive care, and nutrition specialists throughout their cancer care.

Thus, future studies should evaluate the influence of supportive measures such as exercise, nutrition, and symptom management on frailty and should attempt to determine whether frailty is preventable or reversible with such interventions. For patients with a curative plan who are frail, they decision to perform surgery should include a comprehensive evaluation of a patient's performance status (improvement), nutrition, and how they tolerated their neoadjuvant treatments.

## 5 CONCLUSIONS

Frailty is diagnosed in more than one quarter of patients with newly diagnosed PDAC. Frailty is present independently of age or cancer stage at the moment of diagnosis. The diagnosis of frailty is associated with worse survival in patients with who are receiving palliative treatment and in patients with who are receiving curative treatment. Our findings support the establishment of universal frailty screening in patients with newly diagnosed PDAC. Furthermore, this study highlights the importance of interventions to potentially reverse frailty or to prevent its onset in patients with PDAC.

## CONFLICT OF INTEREST

The authors have no conflict of interest to declare.

## AUTHOR CONTRIBUTIONS

An Ngo‐Huang: Project administration; supervision; validation; visualization; writing, reviewing, and editing of original draft. Holly M. Holmes: Conceptualization; data curation; formal analysis; investigation; methodology; project administration; resources; supervision; visualization; writing, reviewing, and editing of original draft. Jude K. A. des Bordes: Conceptualization; data curation; and investigation. Nathan H. Parker: Data curation; investigation; and writing, reviewing, and editing. David Fogelman: Conceptualization; methodology; project administration; supervision; and writing, reviewing, and editing. Maria Q. B. Petzel: Conceptualization; data curation; and writing, reviewing, and editing. Juhee Song: Formal analysis and validation. Eduardo Bruera: Resources; supervision; and writing, reviewing, and editing. Matthew H. G. Katz: Conceptualization; methodology; project administration; resources; supervision; and writing, reviewing, and editing.
